# Outcomes of emergency conversion to general anesthesia during thrombectomy for anterior circulation stroke

**DOI:** 10.1038/s41598-026-39248-2

**Published:** 2026-02-12

**Authors:** Giovanni Merlino, Fedra Kuris, Giacomo Cesco, Paolo Paone, Dario Alimonti, Luca Longhi, Maurizio Passoni, Paolo Gritti, Yan Tereshko, Carolina Gentile, Francesco Janes, Simone Lorenzut, Roberto Marinig, Vladimir Gavrilovic, Cristian Deana, Kyriakos Lobotesis, Berry Stewart, Soma Banerjee, Matteo Foschi, Simona Sacco, Francesco Bax, Thanh N. Nguyen, Massimo Sponza, Gian Luigi Gigli, Lucio D’Anna, Mariarosaria Valente

**Affiliations:** 1 Stroke Unit, Department of Head, Neck, and Neurosciences, Udine University Hospital, Piazzale S. Maria della Misericordia 15, Udine, 33100 Italy; 2 Clinical Neurology, DMED, Udine University Hospital, University of Udine, Udine, Italy; 3 Neurology Unit, Department of Neuroscience, ASST Papa Giovanni XXIII Hospital, Bergamo, Italy; 4 Neurosurgical Intensive Care Unit, Department of Anesthesia and Critical Care Medicine, ASST Papa Giovanni XXIII Hospital, Bergamo, Italy; 5 Division of Vascular and Interventional Radiology, Udine University Hospital, Udine, Italy; 6 Department of Anaesthesia and Intensive Care Health Integrated, Agency of Friuli Centrale, Udine, Italy; 7 Neuroradiology, Department of Imaging, Charing Cross Hospital, Imperial College London, NHS Healthcare Trust, London, UK; 8 Department of Anaesthesia, Charing Cross Hospital, Imperial College Healthcare, London, UK; 9 Department of Stroke and Neuroscience, Charing Cross Hospital, Imperial College London NHS Healthcare Trust, London, UK; 10 Department of Brain Sciences, Imperial College London, London, UK; 11 Department of Biotechnological and Applied Clinical Sciences, University of L’Aquila, L’Aquila, Italy; 12 Hemorrhagic Stroke Research Program, J Philip Kistler Research Center, Department of Neurology, Massachusetts General Hospital, Harvard Medical School, 175 Cambridge Street, Boston, MA 02114 USA; 13 Department of Neurology, Radiology, Boston Medical Center, Boston, MA USA

**Keywords:** Mechanical thrombectomy, Large vessel occlusion, General anesthesia, Local anesthesia, Conscious sedation, Emergency conversion, Functional outcome, Mortality, Diseases, Health care, Medical research, Neurology, Risk factors

## Abstract

The optimal anesthetic strategy during mechanical thrombectomy (MT) for acute ischemic stroke (AIS) remains debated. While general anesthesia (GA) and non-GA approaches are widely used, the impact of emergency conversion (EC) from non-GA to GA is unclear. We evaluated outcomes of patients undergoing EC compared with those managed with primary GA or non-GA. We conducted a multicenter observational study of consecutive anterior circulation large vessel occlusion patients with pre-stroke modified Rankin Scale (mRS) ≤ 2 treated with MT between January 2022 and December 2023 across three centers. Patients were categorized as GA, non-GA, or EC. Inverse probability weighting (IPW) with multivariable adjustment was applied. The primary outcome was 90-day mRS shift; secondary outcomes included pneumonia and 3-month mortality. Among 669 patients, 399 (59.6%) underwent GA, 188 (28.1%) non-GA, and 82 (12.3%) EC. No significant differences were observed in 90-day functional outcomes for EC versus GA (adjusted common OR [acOR] 0.74; 95% CI, 0.48–1.14; *p* = 0.170) or EC versus non-GA (acOR 0.70; 95% CI, 0.40–1.20; *p* = 0.193). Compared with EC, non-GA patients had lower pneumonia risk (acOR 0.17; 95% CI, 0.07–0.45; *p* = 0.001), while GA was associated with reduced 3-month mortality (acOR 0.48; 95% CI, 0.28–0.85; *p* = 0.011). Emergency conversion was not linked to worse functional outcomes compared with GA or non-GA. However, EC was associated with higher pneumonia risk relative to non-GA and increased mortality compared with GA. Larger prospective studies are warranted to clarify the impact of EC during MT.

**Supplementary Information:**

The online version contains supplementary material available at 10.1038/s41598-026-39248-2.

## Introduction

Mechanical thrombectomy (MT), in combination with intravenous thrombolysis (IVT), represents the standard of care for the treatment of acute ischemic stroke (AIS) due to large vessel occlusion (LVO). Recent meta-analyses of randomized controlled trials have failed to demonstrate significant differences in neurological outcomes between general anesthesia (GA) and non-GA techniques, such as local anesthesia (LA) and conscious sedation (CS)^1,2^. More recently, however, the Sedation vs. General Anesthesia for Endovascular Therapy in Acute Ischemic Stroke (SEGA) randomized clinical trial reported a shift toward better 90-day functional outcomes and higher rates of successful reperfusion in patients treated under GA compared with moderate sedation.³ Despite these novel findings, in routine clinical practice the choice of the preferred modality remains largely individualized, taking into account factors such as the patient’s level of cooperation, hemodynamic stability, and stroke severity. Notably, even when MT is initiated under LA or CS, up to 15.6% of patients require emergent conversion to GA, usually due to agitation or clinical deterioration during the procedure^4–7^.

The clinical implications of such emergency conversion (EC) remain poorly characterized, and its impact on the safety and efficacy of MT is still unclear. In this context, our study aimed to compare functional outcomes in patients with anterior circulation LVO undergoing MT, assessing EC against both GA and non-GA approaches.

## Methods

### Study design and participants

**Fig. 1:**
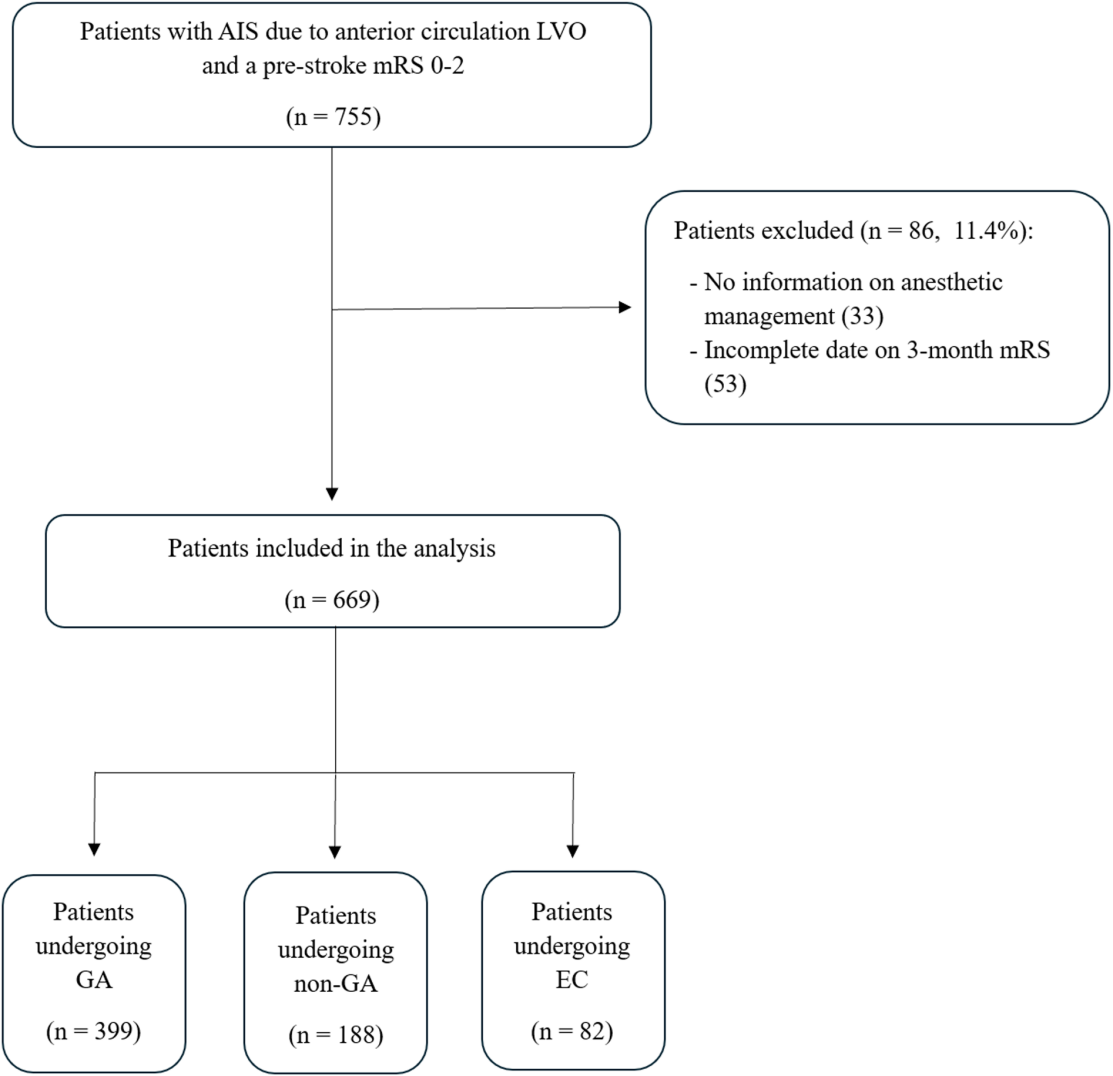
Flowchart of patient selection and inclusion in the study cohort. AIS: acute ischemic stroke; LVO: large vessel occlusion; mRS: modified Rankin Scale; GA: general anesthesia; non-GA: non-general anesthesia; EC: emergency conversion.

This is a multicenter, observational, investigator-initiated, post hoc analysis from prospectively collected data from local registries, which included AIS patients aged 18 years or older consecutively treated with MT for LVO in three comprehensive stroke centers: Udine University Hospital, Udine (Italy); Charing Cross Hospital, Imperial College Healthcare NHS Trust, London (UK); Papa Giovanni XXIII Hospital, Bergamo (Italy) between January 2022 and December 2023. For this analysis, we excluded patients with: (1) pre-stroke mRS > 2; (2) occlusion of the posterior circulation; (3) angiographic evidence of recanalization after intravenous thrombolysis (IVT). Patients with missing data regarding anesthetic management and the primary outcome, i.e., 3-month mRS, were also excluded (Fig. 1). The study was conducted and reported in accordance with the STROBE guidelines.

According to European guidelines, alteplase was used to treat AIS patients with onset of symptoms within 4.5 h and without contraindications or within 9 h if perfusion imaging criteria were met^8^. Additionally, MT was performed in cases with evidence of LVO at CT angiography within 6 h or up to 16–24 h if perfusion criteria were met^9^. The angiographic result of the procedure was assessed on the final digital subtraction angiography (DSA) image series. It was classified according to the modified version of the modified TICI (mTICI) score^10^.

The study was conducted in accordance with the principles outlined in the Declaration of Helsinki. The protocol was approved by the local ethics committee of Udine University Hospital, the coordinating center (Ref. No. IRB: 106–2025). Given its retrospective design and the use of fully anonymized data collected during routine clinical care, the requirement for informed consent was waived in accordance with local regulations.

### Anesthetic management

Since international guidelines do not endorse a specific anesthetic strategy during endovascular treatment, the choice of the preferred anesthetic technique was made collaboratively by the attending anesthesiologist and the interventional neuroradiologist^9,11^.

GA was defined as general anesthesia with endotracheal intubation initiated before the start of the endovascular procedure, while EC was defined as the urgent shift from LA or CS to GA after groin puncture. The decision to convert from LA or CS to GA during the procedure was jointly made by the anesthesiologist, interventional neuroradiologist, and stroke consultant.

With respect to CS and GA, the dosage and combination of the anesthetic drugs were chosen by the attending anesthesiologist. During the procedure, the patient’s hemodynamic state was continuously monitored, and parameters were reported in the medical charts. Following MT, patients who underwent GA, either initiated at the start of the procedure or following EC, were transferred to the intensive care unit (ICU) if extubation was not feasible or considered high-risk; otherwise, they were directly admitted to the Stroke Unit.

### Data collection

All data were prospectively collected, including demographic characteristics (age and sex), relevant medical history (previous stroke or transient ischemic attack, cardiovascular disease, hypertension, atrial fibrillation, diabetes mellitus, hypercholesterolemia, and current smoking), and prior antithrombotic therapy. Atrial fibrillation was classified as known atrial fibrillation (KAF) in patients with a documented history of tachyarrhythmia before the index event, and as atrial fibrillation after stroke (AFAS) in those with a first-ever detection of tachyarrhythmia following the stroke. Baseline clinical parameters included admission systolic blood pressure and blood glucose levels.

Stroke severity was assessed using the National Institutes of Health Stroke Scale (NIHSS) at admission, 24 h after symptom onset, and at discharge, as evaluated by trained vascular neurologists. Pre-stroke functional status was determined using the modified Rankin Scale (mRS). Early ischemic changes in the MCA territory were evaluated on baseline non-contrast CT using the Alberta Stroke Program Early CT Score (ASPECTS)^12^. Stroke etiology was classified according to the Trial of ORG 10,172 in Acute Stroke Treatment (TOAST) criteria^13^.

Procedural data included the site of occlusion, type of thrombectomy device, number of retrieval attempts, presence of secondary embolization, and time from symptom onset to groin puncture (OTG). In patients treated with intravenous alteplase, onset-to-needle time (OTN) was also recorded. We collected data on intra-procedural hemodynamic instability and the specific clinical indications that led to the transition from LC or CS to GA via EC. Hypotensive episodes were defined as a ≥ 25% drop in mean arterial pressure during the procedure and before achieving intracranial reperfusion^14^.

### Study outcomes

The primary outcome was the difference in the 3-month mRS score distribution across the three anesthetic groups (GA, non-GA, and EC), assessed through ordinal shift analysis. The 3-month mRS score was assessed during an in-person follow-up visit or, when not feasible, via telemedicine or telephone interview with the patient or a primary caregiver. The evaluation was conducted by a physician experienced in mRS assessment and blinded to the anesthetic technique used during the thrombectomy procedure.

Secondary clinical study outcomes included moderate-to-severe disability, defined as a 3-month mRS score *≥* 3. Major neurological improvement was defined as an improvement of ≥ 8 points on the NIHSS from baseline or a NIHSS score of 0 or 1 at discharge^15^. Successful recanalization was assessed according to the mTICI and defined as grade 2b, 2c, or 3^16^. Additional secondary outcomes included the occurrence of pneumonia during the hospitalization; symptomatic intracranial hemorrhage (sICH), defined as any ICH on follow-up imaging associated with ⩾4-point worsening on the NIHSS occurring within 7 days of stroke onset^17^; and all causes of 3-month mortality. Pneumonia was defined as the presence of clinical features and radiological findings consistent with a lower respiratory tract infection, as documented in the medical records.

### Statistical analysis

Categorical variables were summarized as counts and percentages, while continuous variables were summarized as medians and interquartile ranges (IQR).

To account for baseline imbalances across anesthesia groups, GA, non-GA, and EC, we applied inverse probability weighting (IPW) based on propensity scores estimated separately for each comparison (GA vs. EC, non-GA vs. EC, and GA vs. non-GA). For each comparison, we selected covariates that were deemed clinically plausible to influence the type of anesthesia and showed relevant imbalance between groups, as assessed by standardized mean differences (SMDs) before weighting. For the GA vs. EC comparison, the IPW model included age, baseline NIHSS score, and ASPECTS. For both the non-GA vs. EC and GA vs. non-GA comparisons, the selected covariates were age, presence of KAF, baseline NIHSS score, TOAST classification, and ASPECTS.

For each of the primary and secondary outcomes, we combined IPW with multivariable adjustment. All weighted models were adjusted for the following covariates, selected a priori based on their clinical relevance to outcomes: age, sex, NIHSS at admission, pre-event mRS, admission glucose level, systolic blood pressure at admission, ASPECT score, receiving IVT, OTG time, site of occlusion, type of occlusion, number of MT passes more than 3, and presence of distal embolization. All variables used in both the IPW models and the multivariable outcome analyses were complete, with no missing data.

Subgroup analyses were conducted for the primary outcome according to age group (≥ 75 vs. < 75 years), sex, baseline NIHSS (≥ 16 vs. < 16), use of bridging IVT, premorbid mRS (0 vs. 1–2), and OTG time (> 360 vs. ≤ 360 min). Interaction terms were tested in the IPW-weighted, adjusted models to assess heterogeneity of the treatment effect across subgroups. P-values for interaction were derived from the coefficient of the interaction term in the weighted and adjusted models.

A sensitivity analysis was performed to evaluate the consistency of the primary outcome across the three participating centers (Udine, London, Bergamo), using separate IPW-weighted and adjusted models stratified by center.

Exploratory analyses were performed within the EC cohort to assess whether outcomes differed according to the primary trigger for emergency conversion. Given the small number of patients in individual non-agitation categories, conversion triggers were grouped into agitation-related versus other indications (including procedural complications, vomiting or aspiration, respiratory failure, and other clinical conditions). Comparisons between groups were performed using Fisher’s exact test for binary outcomes and the Wilcoxon rank-sum test for ordinal outcomes. Outcome comparisons were descriptive and exploratory in nature.

To account for multiple comparisons across the prespecified anesthesia group contrasts, p-values for the primary and secondary outcomes derived from the IPW-weighted and multivariable-adjusted analyses were additionally adjusted using the Benjamini–Hochberg false discovery rate procedure.

All analyses were performed using R software, version 4.2. Statistical significance was set at a p-value < 0.05.

## Results

A total of 669 AIS patients due to anterior circulation LVO who underwent MT were included in the analysis. Among them, 399 (59.6%) were treated under GA, 188 (28.1%) under non-GA modalities, and 82 (12.3%) underwent EC during the endovascular procedure (Fig. 1).

Demographic and clinical characteristics are presented in Table 1, while Table 2 summarizes neuroradiological and procedural features across the three anesthetic groups. Overall, after applying IPW, a good balance was achieved for almost all variables of interest.

**Table 1:** Baseline demographic and clinical characteristics according to anesthetic strategy (GA, non-GA, and EC), before and after inverse probability weighting.

	GA (*N* = 399)	non-GA (*N* = 188)	EC (*N* = 82)	GA vs. EC SMD unweighted	GA vs. EC SMD weighted	non-GA vs. EC SMD unweighted	non-GA vs. EC SMD weighted	GA vs. non-GA SMD unweighted	GA vs. non-GA SMD weighted
Age, years [median (IQR)]	71 (61–81)	80 (72-84.2)	68.5 (57–79)	0.1304	0.0288	0.6315	0.0384	0.5280	0.1118
Female sex [n, (%)]	191 (47.9)	102 (54.3)	43 (52.4)	0.0457	0.0234	0.0182	0.0081	0.0639	0.0171
Hypertension [n, (%)]	222 (55.6)	120 (63.8)	43 (52.4)	0.0320	0.0171	0.1139	0.0442	0.0819	0.0171
DM [n, (%)]	45 (11.3)	32 (17)	14 (17.1)	0.0579	0.0176	0.0005	0.0130	0.0574	0.0256
Hypercholesterolemia [n, (%)]	109 (27.3)	55 (29.3)	30 (36.6)	0.0927	0.1114	0.0733	0.1043	0.0194	0.0027
KAF [n, (%)]	85 (21.3)	67 (35.6)	10 (12.2)	0.0911	0.0825	0.2344	0.0688	0.1434	0.0172
AFAS [n, (%)]	27 (6.8)	28 (14.9)	6 (7.3)	0.0055	0.0079	0.0758	0.0479	0.0813	0.0026
CVD [n, (%)]	48 (12)	22 (11.7)	16 (19.5)	0.0748	0.0838	0.0781	0.0600	0.0033	0.0015
PE [n, (%)]	29 (7.3)	7 (3.7)	4 (4.9)	0.0239	0.0133	0.0115	0.0025	0.0354	0.0377
TIA/IS [n, (%)]	61 (15.3)	31 (16.5)	14 (17.1)	0.0178	0.0342	0.0058	0.0413	0.0120	0.0056
Smoker [n, (%)]	67 (16.8)	18 (9.6)	15 (18.3)	0.0150	0.0010	0.0872	0.0392	0.0722	0.0213
Pre-event mRS	0 (0–1)	0 (0–1)	0 (0–0)	0.0659	0.0187	0.1157	0.0002	0.0499	0.0434
Baseline NIHSS [median (IQR)]	18 (13.5–22)	15 (10.7–20)	17 (11-20.7)	0.1913	0.0594	0.1771	0.0215	0.4125	0.0932
Glucose at admission (mg/dl) [median (IQR)]	122 (108–142)	126 (104–153)	127 (110–153)	0.1806	0.1473	0.1247	0.0092	0.0592	0.0661
SBP at admission (mmHg) [median (IQR)]	148 (130.5–169)	150 (140–170)	145 (130–167)	0.0578	0.5440	0.2462	0.3104	0.1800	0.1387
***Toast classification***:									
LAA [n, (%)]	79 (19.8)	25 (13.3)	17 (20.7)	0.0093	0.0059	0.0743	0.0037	0.0650	0.0034
CE [n, (%)]	103 (25.8)	97 (51.6)	14 (17.1)	0.0874	0.0777	0.3452	0.0397	0.2578	0.0126
Other cause [n, (%)]	54 (13.5)	13 (6.9)	14 (17.1)	0.0354	0.0309	0.1016	0.0010	0.0662	0.0036
Undetermined cause [n, (%)]	163 (40.9)	53 (28.2)	37 (45.1)	0.0427	0.0409	0.1693	0.0349	0.1266	0.0123
***Admission therapy***:									
Anticoagulation [n, (%)]	30 (7.5)	34 (18.1)	7 (8.5)	0.0102	0.0215	0.0955	0.0461	0.1057	0.0730
Antiplatelets [n, (%)]	69 (17.3)	50 (26.6)	20 (24.4)	0.0710	0.0556	0.0221	0.0546	0.0930	0.0481

SMD: standardized mean difference; IQR: interquartile range; GA: general anesthesia; non-GA: non-general anesthesia; EC: emergency conversion; DM: diabetes mellitus; KAF: known atrial fibrillation; AFAS: atrial fibrillation after stroke; CVD: cardiovascular disease; PE: pulmonary embolism; TIA/IS: transient ischemic attack/ischemic stroke; mRS: modified Rankin Scale; NIHSS: National Institutes of Health Stroke Scale; SBP: systolic blood pressure; LAA: large artery atherosclerosis; CE: cardioembolism.

**Table 2:** Neuroradiological and procedural characteristics according to anesthetic strategy (GA, non-GA, and EC), before and after inverse probability weighting.

	GA (*N* = 399)	non-GA (*N* = 188)	EC (*N* = 82)	GA vs. EC SMD unweighted	GA vs. EC SMD weighted	non-GA vs. EC SMD unweighted	non-GA vs. EC SMD weighted	GA vs. non-GA SMD unweighted	GA vs. non-GA SMD weighted
ASPECT score on admission [median (IQR)]	10 (8–10)	10 (9–10)	10 (9–10)	0.1545	0.0107	0.2395	0.0211	0.4105	0.0127
IVT [n, (%)]	233 (58.4)	111 (59)	47 (57.3)	0.0108	0.0247	0.0173	0.0553	0.0065	0.0273
OTN (min) [median (IQR)]	130 (105–162)	135 (105–162)	140 (105-175.8)	0.0228	0.0279	0.0361	0.0488	0.0532	0.1081
OTG (min) [median (IQR)]	218 (176.5–265)	210 (160-263.8)	220 (175-263.8)	0.0400	0.0720	0.0377	0.1410	0.0717	0.1129
Hypotension during MT [n, (%)]	77 (19.3)	20 (10.6)	14 (17.1)	0.0223	0.0256	0.0643	0.0743	0.0866	0.0799
***Site occlusion***:									
M1 [n, (%)]	182 (45.6)	100 (53.2)	32 (39)	0.0659	0.0649	0.1417	0.1338	0.0758	0.0732
M2 [n, (%)]	107 (26.8)	43 (22.9)	29 (35.4)	0.0855	0.0805	0.1249	0.2222	0.0394	0.0970
Intracranial ICA [n, (%)]	49 (12.3)	22 (11.7)	10 (12.2)	0.0009	0.0132	0.0049	0.0343	0.0058	0.0066
Tandem occlusion [n, (%)]	61 (15.3)	23 (12.2)	11 (13.4)	0.0187	0.0024	0.0118	0.0541	0.0305	0.0304
***MT technique***:									
Aspiration [n, (%)]	103 (25.8)	47 (25.1)	24 (29.3)	0.0345	0.0024	0.0413	0.0124	0.0068	0.0478
Stent retriever [n, (%)]	224 (56.1)	41 (21.9)	48 (58.5)	0.0240	0.0153	0.3661	0.3662	0.3422	0.3363
Combined technique [n, (%)]	57 (14.3)	92 (49.2)	9 (11)	0.0331	0.0332	0.3822	0.3645	0.3491	0.3648
Permanent Stenting [n, (%)]	15 (3.8)	7 (3.7)	1 (1.2)	0.0254	0.0223	0.0252	0.0101	0.0002	0.0193
Number of passes more than 3 [n, (%)]	109 (27.3)	65 (34.6)	21 (25.6)	0.0171	0.0062	0.0896	0.1470	0.0726	0.1174
Distal embolization [n, (%)]	14 (7.4)	27 (6.8)	3 (3.7)	0.0311	0.0290	0.0379	0.0525	0.0068	0.0067

SMD: standardized mean difference; IQR: interquartile range; GA: general anesthesia; non-GA: non-general anesthesia; EC: emergency conversion; ASPECT: Alberta Stroke Program Early CT; IVT: intravenous thrombolysis; OTN: onset-to-needle; OTG: onset-to-groin; MT: mechanical thrombectomy; ICA: internal carotid artery.

EC was most frequently triggered by agitation (*n* = 67, 81.7%), followed by procedural complications (*n* = 4, 4.9%), vomiting or aspiration (*n* = 4, 4.9%), respiratory failure (*n* = 2, 2.4%), and other clinical conditions (*n* = 5, 6.1%).

### Primary outcome

**Fig. 2:**
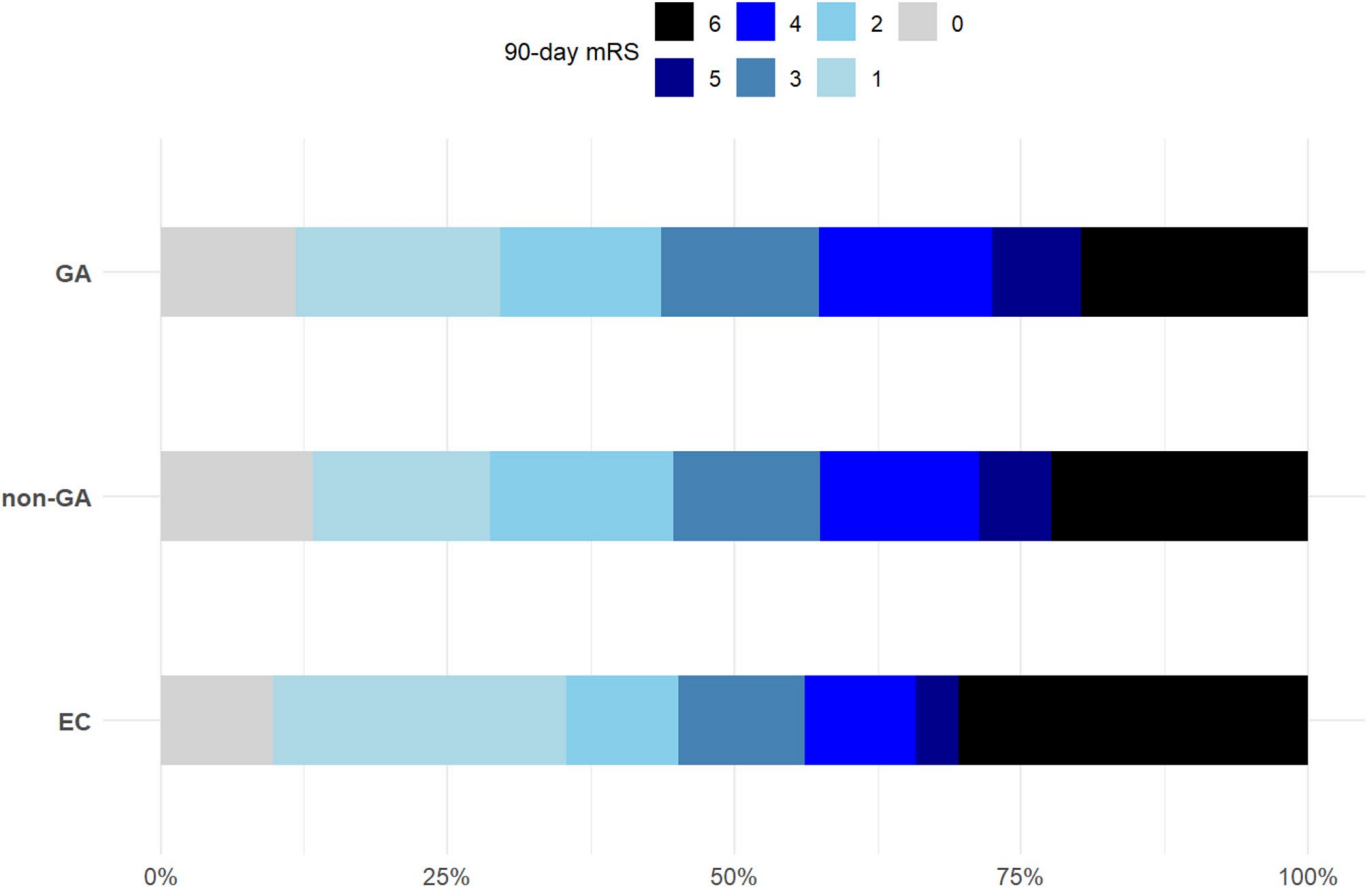
Distribution of 90-day modified Rankin Scale scores across anesthetic groups (GA, non-GA, and EC). mRS: modified Rankin Scale; GA: general anesthesia; non-GA: non-general anesthesia; EC: emergency conversion.

Unweighted and weighted shift analyses showed no significant differences in the 3-month mRS distribution among patients treated with GA, non-GA, or EC. Results were confirmed by models that combined IPW and multivariable adjustment for several covariates (Tables 3 and 4). Across all comparisons, EC was not independently associated with a worse 3-month mRS distribution (Fig. 2).

**Table 3:** Distribution of 90-day modified Rankin scale scores and ordinal logistic regression analyses comparing emergency conversion (EC) versus primary general anesthesia (GA).

Outcome (3-month mRS)		EC, *n* (%)	GA, *n* (%)
mRS 0		8 (9.8)	47 (11.8)
mRS 1		21 (25.6)	71 (17.8)
mRS 2		8 (9.8)	56 (14.0)
mRS 3		9 (11.0)	55 (13.8)
mRS 4		8 (9.8)	60 (15.0)
mRS 5		3 (3.7)	31 (7.8)
mRS 6		25 (30.5)	79 (19.8)

Unweighted mRS shift (univariate)			
*Predictors*	*cOR*	*95% CI*	*p*
Type of anesthesia			
EC	1^*^		
GA	0.90	0.90–2.08	0.636

Weighted mRS shift (univariate)			
*Predictors*	*cOR*	*95% CI*	*p*
Type of anesthesia			
EC	1^*^		
GA	0.73	0.48–1.11	0.139

Weighted mRS shift (multivariate)^1^			
*Predictors*	*acOR*	*95% CI*	*p (unadj); p (FDR-adj)*
Type of anesthesia			
EC	1^*^		
GA	0.74	0.48–1.14	0.170; 0.193

cOR: common odds ratio; acOR: adjusted common odds ratio; 95% CI: 95% confidence interval. EC: emergency conversion; GA: general anesthesia; mRS: modified Rankin Scale. False discovery rate (FDR)-adjusted p-values were calculated using the Benjamini–Hochberg procedure. *Reference category. ^1^Multivariable models were adjusted for age, sex, baseline National Institutes of Health Stroke Scale score, pre-event modified Rankin Scale score, admission glucose level, admission systolic blood pressure, Alberta Stroke Program Early CT Score, intravenous thrombolysis, onset-to-groin time, site of occlusion, procedural technique, number of thrombectomy passes (> 3), and distal embolization.

**Table 4:** Distribution of 90-day modified Rankin scale scores and ordinal logistic regression analyses comparing emergency conversion (EC) versus non-general anesthesia (non-GA).

Outcome (3-month mRS)		EC, *n* (%)	non-GA, *n* (%)
mRS 0		8 (9.8)	25 (13.3)
mRS 1		21 (25.6)	29 (15.4)
mRS 2		8 (9.8)	30 (16.0)
mRS 3		9 (11.0)	24 (12.8)
mRS 4		8 (9.8)	26 (13.8)
mRS 5		3 (3.7)	12 (6.4)
mRS 6		25 (30.5)	42 (22.3)

Unweighted mRS shift (univariate)			
*Predictors*	*cOR*	*95% CI*	*p*
Type of anesthesia			
EC	1^*^		
non-GA	0.92	0.58–1.46	0.726

Weighted mRS shift (univariate)			
*Predictors*	*cOR*	*95% CI*	*p*
Type of anesthesia			
EC	1^*^		
non-GA	1.16	0.74–1.84	0.514

Weighted mRS shift (multivariate)^1^			
*Predictors*	*acOR*	*95% CI*	*p (unadj); p (FDR-adj)*
Type of anesthesia			
EC	1^*^		
non-GA	0.70	0.40–1.20	0.193; 0.193

cOR: common odds ratio; acOR: adjusted common odds ratio; 95% CI: 95% confidence interval; EC: emergency conversion; non-GA: non-general anesthesia; mRS: modified Rankin Scale. False discovery rate (FDR)-adjusted p-values were calculated using the Benjamini–Hochberg procedure. *Reference category. ^1^Multivariable models were adjusted for age, sex, baseline National Institutes of Health Stroke Scale score, pre-event modified Rankin Scale score, admission glucose level, admission systolic blood pressure, Alberta Stroke Program Early CT Score, intravenous thrombolysis, onset-to-groin time, site of occlusion, procedural technique, number of thrombectomy passes (> 3), and distal embolization.

### Secondary outcomes

Results from the weighted and adjusted logistic regression model showed that patients undergoing EC had comparable 3-month disability outcomes to those treated with either GA or non-GA techniques. The incidence of sICH was also similar across groups. The decision to convert from non-GA to GA via EC did not adversely affect neurological outcomes or the odds of successful recanalization after MT. However, EC patients had a significantly higher risk of pneumonia during hospitalization compared to those treated with non-GA. Despite this, the 3-month mortality rate among EC patients was not significantly higher overall (acOR: 0.49; 95% CI: 0.21–1.13; *p* = 0.095). Notably, 3-month mortality was significantly lower in the GA group compared to EC. In adjusted analysis, GA was associated with a 52% reduction in the odds of death at 3 months compared to EC (Supplemental Tables 1 and 2).

### Subgroup analysis

Results of the subgroup analyses for the primary outcome, based on the IPW-weighted and adjusted model, are presented in Supplemental Fig. 1. No significant interactions were detected across the predefined subgroups (Supplemental Fig. 1).

### Sensitivity analysis

The sensitivity analysis confirmed the consistency of the primary outcome across the three participating centers, using separate IPW-weighted and adjusted models stratified by center. No significant interaction was found between center and anesthetic group (p for interaction > 0.05), supporting the robustness of the overall findings (Supplemental Fig. 2).

### Exploratory analysis

In exploratory inferential analyses restricted to the EC cohort, no statistically significant differences were observed between patients converted due to agitation and those converted for other indications with respect to the primary and secondary outcomes; detailed results are reported in the Supplemental Table 3.

## Discussion

This multicenter observational study demonstrated that EC to GA during MT for anterior circulation LVO was not associated with worse functional outcomes at 90 days when compared to either primary GA or non-GA (LA or CS), in patients who were functionally independent prior to stroke. However, EC was linked to a higher risk of post-procedural pneumonia compared to non-GA approaches, and increased 3-month mortality compared to patients treated under primary GA.

The optimal anesthetic strategy for MT in anterior circulation LVO remains a topic of debate. A meta-analysis by Campbell et al. reported that GA increased successful recanalization and functional recovery without affecting hemorrhagic complications or mortality^18^. More recently, the Sedation vs. General Anesthesia for Endovascular Therapy in Acute Ischemic Stroke (SEGA) randomized clinical trial reported a shift toward improved 90-day functional outcomes and higher rates of successful reperfusion in patients treated under planned GA compared with moderate sedation^3^. Importantly, however, SEGA compared predefined anesthetic strategies under controlled trial conditions and excluded patients with severe agitation, airway compromise, or early clinical instability. In this context, EC from non-GA to GA was treated as a crossover event rather than as a distinct clinical entity, and its specific clinical implications were not directly addressed. A recent multicenter study from the German Stroke Registry (GSR-ET) found similar recanalization rates but better functional outcomes with non-GA in minor strokes (NIHSS < 6)^19^. Since non-GA is commonly used in less severe cases, clarifying the clinical impact of EC to GA is essential.

In our study baseline characteristics differed substantially between patients treated under GA and those managed with non-GA, reflecting real-world, clinically driven anesthetic decision-making rather than random allocation. Patients treated without GA were markedly older and exhibited a higher burden of cardiovascular comorbidities, including a greater prevalence of atrial fibrillation and cardioembolic stroke etiology, factors that may have discouraged the upfront use of general anesthesia. In contrast, patients managed under GA presented with greater stroke severity at admission, as reflected by higher baseline NIHSS scores, and slightly less favorable imaging profiles, as suggested by differences in ASPECTS. Taken together, these baseline differences likely influenced the initial anesthetic strategy and underscore the potential for confounding by indication when comparing outcomes across anesthesia groups in observational studies.

To date, data specifically addressing EC patients in large studies and RCTs comparing CS versus GA remain scarce. This gap may stem from the methodological challenges associated with EC, which is often considered either a treatment crossover (from non-GA to GA) or a protocol deviation, leading to its inclusion under varying analytic frameworks such as intention-to-treat, as-treated, or per-protocol analyses. Nonetheless, EC represents a distinct rescue intervention, and while the number of patients undergoing EC is not negligible, it remains relatively limited in most available cohorts. The French AMETIS trial group made an effort to address this knowledge gap by conducting an ancillary analysis focusing on patients randomized to the CS arm and explicitly investigating “conversion with intubation” as one of the exploratory secondary endpoints. EC emerged as a significant predictor of poor functional outcome (aOR: 32, 95% CI, 1.11–96.4; *p* = 0.01). Notably, the wide confidence interval reflects limited statistical precision, likely due to the small number of events, and suggests that this finding should be interpreted with caution^7^. The retrospective single-center study by Flottmann et al. analyzed 254 patients with anterior circulation LVO treated with MT between 2015 and 2018, of whom 25 (9.8%) required EC to GA. The study found no significant differences in rates of successful recanalization or functional independence between EC patients and those treated under either primary GA or non-GA modalities^14^. Similar findings were reported in the single-center study by Chen et al., who performed a post hoc analysis of data from the KEEP SIMPLEST study. Among 160 patients, 20 (12.5%) required emergent intubation during the procedure. After multivariable logistic and ordinal regression analyses, emergency conversion was not significantly associated with worse functional outcomes compared to patients who remained under primary procedural sedation^20^. Notably, this study included in the EC group patients who were converted to GA prior to groin puncture. These individuals would be classified as primary GA recipients in the analyses by Flottmann et al. and in our own. Our multicenter study is consistent with these earlier findings. Both unweighted and weighted analyses, after multiple adjustments, revealed that EC was not associated with an increased odds of worse functional outcome at 3 months, as assessed by the full mRS distribution. Consistent results were observed when the mRS was analyzed as a dichotomized outcome.

No significant interactions were observed in the subgroup analysis for the primary outcome. In the EC vs. GA comparison, patients with higher stroke severity (NIHSS ≥ 16) showed a trend toward better outcomes with GA compared to EC (acOR: 0.52; 95% CI, 0.30–0.91), although the interaction p-value was not significant (*p* = 0.150). Similarly, in the EC vs. non-GA comparison, patients who received IVT showed a trend favoring non-GA over EC (acOR: 0.42; 95% CI, 0.19–0.93), again without a significant interaction (*p* = 0.354). These findings suggest that the impact of anesthetic strategy relative to EC was broadly consistent across subgroups, with no evidence of effect modification. Results were also consistent in the center-based sensitivity analysis, which showed no significant heterogeneity across sites (interaction *p* = 0.574 for EC vs. GA; *p* = 0.798 for EC vs. non-GA), supporting the robustness of the overall findings.

Agitation during MT is the leading cause of EC to GA. In the ancillary analysis of the AMETIS trial, intraprocedural agitation was significantly associated with a higher rate of conversion with intubation compared to non-agitated patients, 21% versus 5%^7^. Similarly, Flottmann et al. reported that emergency conversion was primarily triggered by agitation or excessive movement in 72% of cases^14^. In an Italian retrospective study, agitation or excessive movement was identified as the cause of emergency conversion in 14 out of 20 patients initially managed with non-GA^21^. In our cohort, the vast majority of emergency conversions, 81.7%, were due to intraprocedural agitation, emphasizing that difficulty in maintaining patient cooperation under non-GA conditions may lead to EC. By contrast, conversion due to medical complications such as vomiting, aspiration, or respiratory failure was relatively rare. In an exploratory analysis, no statistically significant differences in outcomes were observed between patients undergoing EC due to agitation and those converted for other indications.

Intubation is a well-established risk factor for pneumonia^22^. A recent meta-analysis by Jia et al. reported that AIS patients undergoing MT had nearly 1.5 times greater odds of developing pneumonia when treated under GA compared to CS^1^. In our cohort, patients treated with non-GA had 83% lower odds of developing pneumonia compared to those who were emergently converted from LA or CS to GA. Despite this difference, 3-month mortality rates did not differ significantly between EC and non-GA groups. In contrast, patients treated with GA from the outset had significantly lower 3-month mortality compared to those who underwent EC. Further studies are warranted to confirm these preliminary findings.

Our study has several strengths. To date, it represents the largest cohort of EC patients reported in the literature. Although observational in nature, the use of IPW enabled pseudo-randomization, which helped mitigate bias arising from demographic and clinical factors that influence anesthetic management. Furthermore, an IPW-weighted and adjusted approach was applied by performing multivariable adjustment on the weighted models, thereby enhancing the reliability of the findings. Nonetheless, as a post-hoc observational analysis, the possibility of residual confounding cannot be excluded despite adjustment for multiple covariates. In particular, although baseline measured characteristics of patients undergoing EC were broadly comparable to those treated under primary GA, EC may still identify a subgroup of patients with unmeasured clinical vulnerability or intra-procedural instability not fully captured by baseline variables, which could have influenced mortality outcomes. A further limitation of our study is the lack of detailed information regarding the type and dosage of anesthetic agents used during GA and EC. In addition, granular procedural data—including puncture-to-final-recanalization time, depth of sedation, sedation escalation strategies, and the sequence of sedative agents administered prior to emergency conversion—were not systematically collected. This prevents any assessment of the potential impact of procedural timing or optimization of sedation depth on patient outcomes. Similarly, data on post-procedural management were not systematically available, including timing of extubation, need for additional neuroimaging due to delayed neurological assessment under GA, and differences in post-EVT resource utilization or healthcare costs between GA and non-GA strategies. Future studies should systematically collect and analyze these data to clarify whether variations in anesthetic management and sedation escalation strategies, as well as post-procedural management and resource utilization, may influence clinical results. Moreover, the relatively small size of the EC group, despite being the largest reported to date, may have limited statistical power for selected comparisons. Finally, pneumonia was identified retrospectively from medical records rather than through a centralized adjudication using predefined diagnostic criteria, and some degree of outcome misclassification cannot be excluded.

In conclusion, our study suggests that initiating MT under non-GA may be a reasonable strategy in selected patients, including those who might eventually require EC during the procedure. Since EC was not associated with significantly worse functional outcomes compared to either GA or non-GA, starting with a non-GA approach could be considered when no clear contraindications to conscious sedation or local anesthesia are present. Nevertheless, EC was associated with a higher risk of pneumonia compared to non-GA and increased 3-month mortality compared to GA, underscoring the need for cautious patient selection, vigilant intra-procedural monitoring, and optimal airway management. Further studies are warranted to confirm these findings.

## Supplementary Information

Below is the link to the electronic supplementary material.

Supplementary Material 1

## Data Availability

The corresponding author can provide the datasets upon reasonable request.

## Declarations

### Guarantor

G.M.
