# Supplementary material for: Outcomes of emergency conversion to general anesthesia during thrombectomy for anterior circulation stroke
Source: Sci Rep. 2026 Feb 12;16:8450. doi: 10.1038/s41598-026-39248-2 (PMC12972312; doi:10.1038/s41598-026-39248-2)
Supplement: Supplementary file 1 — Supplementary Material 1 [file 41598_2026_39248_MOESM1_ESM.pdf]

Supplemental Materials

Table S1. Inverse probability weighted and multivariable-adjusted logistic regression analyses of secondary outcomes comparing emergency conversion (EC) versus general anesthesia (GA)

	**3-month mRS ≥3**				**Major neurological improvement**				**Successful recanalization**			
Predictors	*acOR*	*95% CI*	$p^{\dagger}$	$p^{\ddagger}$	*acOR*	*95% CI*	$p^{\dagger}$	$p^{\ddagger}$	*acOR*	*95% CI*	$p^{\dagger}$	$p^{\ddagger}$
Type of anesthesia												
EC	1*				1*				1*			
GA	0.79	0.46-1.36	0.393	0.540	1.19	0.71-2.01	0.501	0.501	1.89	0.89-3.99	0.097	0.097
	**Pneumonia**				**sICH**				**3-month mortality**			
Predictors	*acOR*	*95% CI*	$p^{\dagger}$	$p^{\ddagger}$	*acOR*	*95% CI*	$p^{\dagger}$	$p^{\ddagger}$	*acOR*	*95% CI*	$p^{\dagger}$	$p^{\ddagger}$
Type of anesthesia												
EC	1*				1*				1*			
GA	0.94	0.50-1.77	0.839	0.839	0.41	0.12-1.48	0.174	0.348	0.48	0.28-0.85	**0.011**	**0.022**

acOR: adjusted common odds ratio; 95% CI: 95% confidence interval; EC: emergency conversion; GA: general anesthesia; mRS: modified Rankin Scale; sICH: symptomatic intracranial hemorrhage.
False discovery rate (FDR)-adjusted p-values were calculated using the Benjamini–Hochberg procedure.
*Reference category. †p-value unadjusted. ‡p-value FDR-adjusted.
Multivariable models were adjusted for age, sex, baseline National Institutes of Health Stroke Scale score, pre-event modified Rankin Scale score, admission glucose level, admission systolic blood pressure, Alberta Stroke Program Early CT Score, intravenous thrombolysis, onset-to-groin time, site of occlusion, procedural technique, number of thrombectomy passes (>3), and distal embolization.

Table S2. Inverse probability weighted and multivariable-adjusted logistic regression analyses of secondary outcomes comparing emergency conversion (EC) versus non-general anesthesia (non-GA)

	3-month mRS ≥3				Major neurological improvement				Successful recanalization			
Predictors	*acOR*	*95% CI*	$p^{\dagger}$	$p^{\ddagger}$	*acOR*	*95% CI*	$p^{\dagger}$	$p^{\ddagger}$	*acOR*	*95% CI*	$p^{\dagger}$	$p^{\ddagger}$
Type of anesthesia												
EC	1^{*}				1^{*}				1^{*}			
non-GA	0.79	0.37-1.69	0.540	0.540	1.33	0.65-2.73	0.428	0.501	2.52	0.86-7.41	0.092	0.097
	**Pneumonia**				**sICH**				**3-month mortality**			
Predictors	*acOR*	*95% CI*	$p^{\dagger}$	$p^{\ddagger}$	*acOR*	*95% CI*	$p^{\dagger}$	$p^{\ddagger}$	*acOR*	*95% CI*	$p^{\dagger}$	$p^{\ddagger}$
Type of anesthesia												
EC	1^{*}				1^{*}				1^{*}			
non-GA	0.17	0.07-0.45	**0.001**	**0.002**	0.63	0.11-3.67	0.611	0.611	0.49	0.21-1.13	0.095	0.095

acOR: adjusted common odds ratio; 95% CI: 95% confidence interval; EC: emergency conversion; non-GA: non-general anesthesia; mRS: modified Rankin Scale; sICH: symptomatic intracranial hemorrhage.

False discovery rate (FDR)-adjusted p-values were calculated using the Benjamini–Hochberg procedure.

*Reference category. †p-value unadjusted. ‡p-value FDR-adjusted.

Multivariable models were adjusted for age, sex, baseline National Institutes of Health Stroke Scale score, pre-event modified Rankin Scale score, admission glucose level, admission systolic blood pressure, Alberta Stroke Program Early CT Score, intravenous thrombolysis, onset-to-groin time, site of occlusion, procedural technique, number of thrombectomy passes (>3), and distal embolization.

Table S3. Exploratory comparison of outcomes in emergency conversion patients by conversion trigger (agitation vs other indications)

	**Median 3-month mRS (IQR)**		**p**
Agitation	3 (1-6)		
Other indications	4 (1-6)		0.892
**Outcome**	**Agitation (n = 67)**	**Other indications (n = 15)**	**p**
3-month mRS ≥3, n (%)	37 (55.2)	8 (53.3)	1.000
Major neurological improvement, n (%)	34 (50.7)	6 (40.0)	0.571
Successful recanalization, n (%)	61 (91.0)	13 (86.7)	0.634
Pneumonia, n (%)	12 (17.9)	4 (26.7)	0.477
sICH, n (%)	3 (4.5)	2 (13.3)	0.255
3-month mortality, n (%)	20 (29.9)	5 (33.3)	0.766

IQR: interquartile range; EC: emergency conversion; mRS: modified Rankin Scale; sICH: symptomatic intracranial hemorrhage.
Exploratory, unadjusted analyses restricted to the EC cohort. The distribution of 3-month modified Rankin Scale (mRS) scores was compared using the Wilcoxon rank-sum test, and binary outcomes were compared using Fisher's exact test. No adjustment for multiple comparisons was applied.

Figure S1. Subgroup analyses of 90-day modified Rankin Scale shift comparing emergency conversion (EC) versus general anesthesia (GA) (top) and versus non-general anesthesia (non-GA) (bottom).

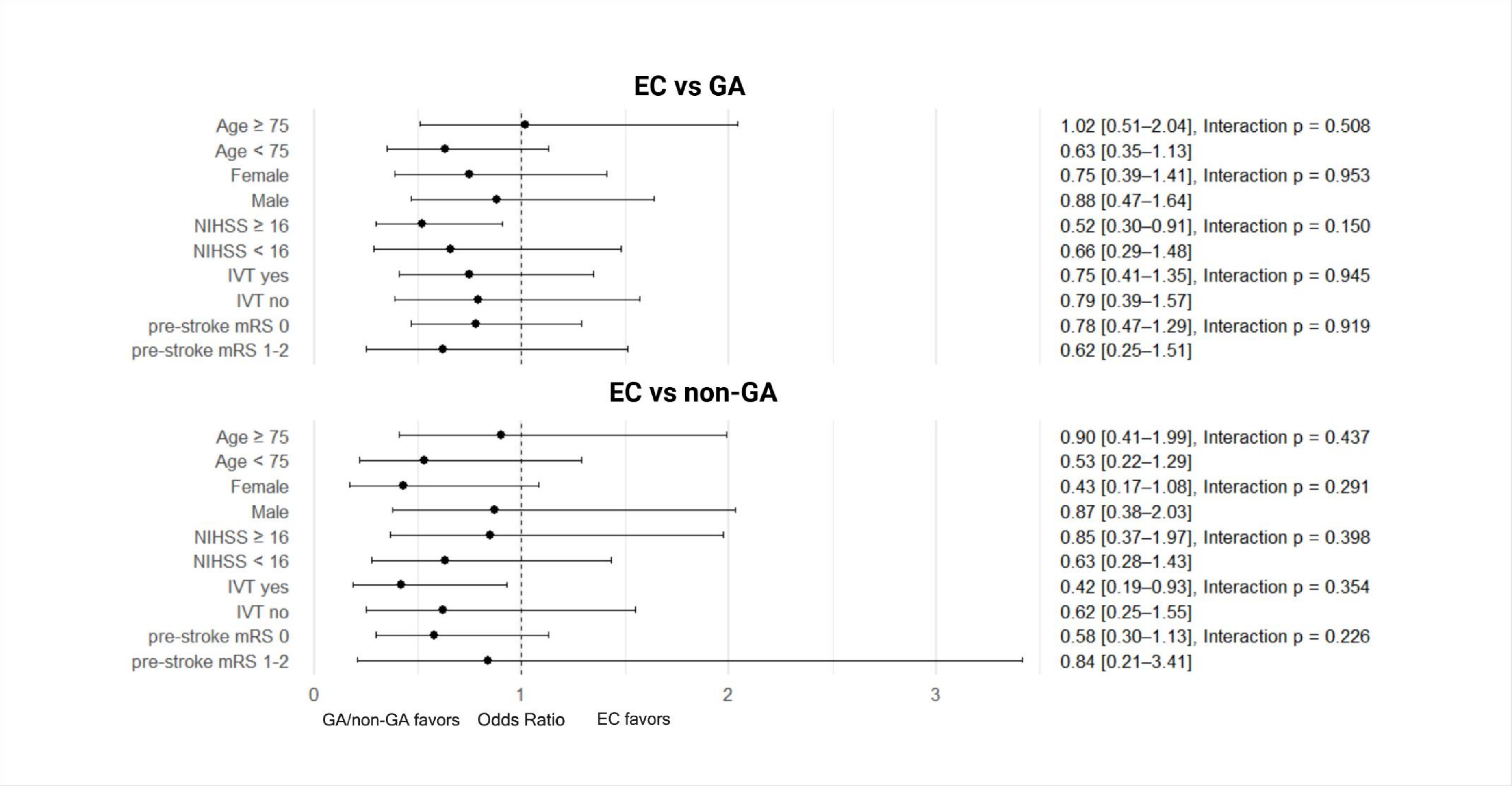

EC: emergency conversion; GA: general anesthesia; non-GA: non-general anesthesia; NIHSS: National Institutes of Health Stroke Scale; IVT: intravenous thrombolysis; mRS: modified Rankin Scale.
EC is the reference category. Odds ratios <1 indicate a better functional outcome (lower mRS) in GA or non-GA compared to EC.

Figure S2. Center-stratified analyses of 90-day modified Rankin Scale shift comparing emergency conversion (EC) versus general anesthesia (GA) (top) and versus non-general anesthesia (non-GA) (bottom).

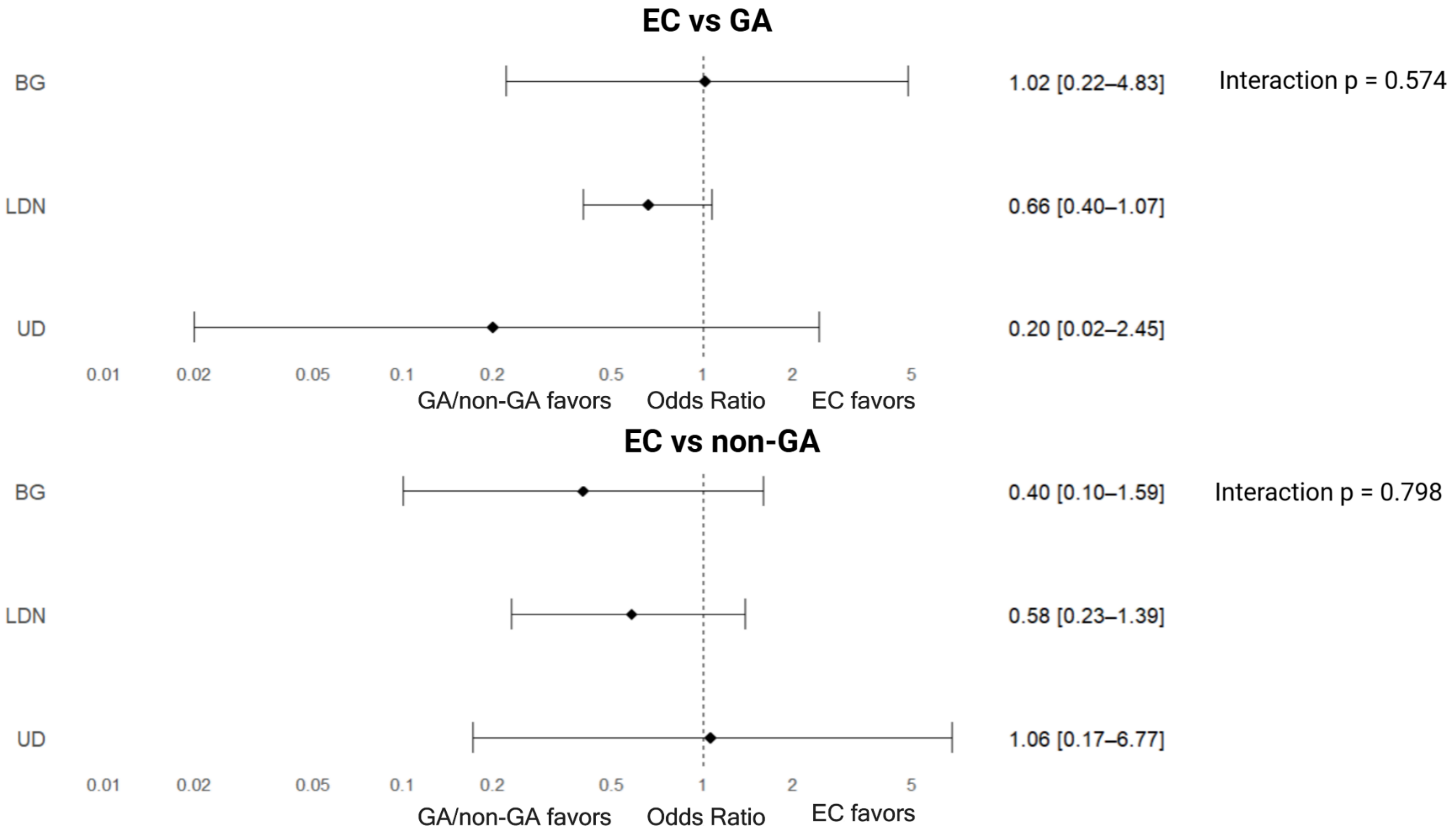

EC: emergency conversion; GA: general anesthesia; non-GA: non-general anesthesia; BG: Bergamo; LDN: London; UD: Udine.
EC is the reference category. Odds ratios <1 indicate a better functional outcome (lower mRS) in GA or non-GA compared to EC.
